# Operation for Club Foot

**Published:** 1843-11

**Authors:** 


### OPERATION FOR CLUB FOOT.

The operation for this deformity has recently been successfully performed by Dr. A. C. Hensley, of Versailles. We extract the following account of it from his letter dated August 23d:

"The patient, Mr. Frazer, is about sixteen years of age. About thirteen years since he had an attack of fever, very soon after which the great toe became gradually extended; not many months after, elevation of the heel began to take place, and from this time he has been walking on the extremities of the metatarsal bones. In the last four months the foot has grown rapidly worse. At present his state is as follows: After the most minute examination I find his general health as good as could be desired. As relates to the condition of the affected member I find the cutaneous sensibility quite as lively in it as the other; motions performed with equal *celerity.* The degree of atrophy is considerable, amounting to *a marked contrast* in the size of the two members; the muscles however are firm. The distortion of the foot is caused by the contraction of the muscles which terminate in the tendo achillis, the tibialis posticus and the extensors of the great and littles toes. When laid on a bed the heel of the affected foot cannot be brought as low as the heel of the sound foot, by full two inches; the plantar aspect of the foot very *deep* or concave; the extension of the great and little toes so great as to elevate them considerably out of their natural position. The foot is slightly though not decidedly inclined inwards. There is no anchylosis but a very considerable degree of displacement between the individual bones making up the tarsus. The tarsal-joint is free in its movements until prevented by the contraction of the tibialis posticus. This in short is a case of club-foot of that variety called *pes equinus,* of the second degree, very well advanced.

"On the 22d of June, I operated by dividing the tendo achillis and the tendons of the extensors of the great and little toes, on the plan of Mr. Whipple of Plymouth, except that the tendons were divided transversely instead of obliquely. The heel descended three quarters of an inch immediately, and the toes came to their normal position. The incisions were at once closed with adhesive strips, and Professor Mutter's stretching apparatus was applied by whichan attempt was made to bring the heel gradually but completely down. This was accomplished, as well as re-union of the external wound, by the eighth day after the operation. From this time the foot and leg were kept stationary with the view of promoting the organization of the intermedium by which the divided tendons were to gain length and be re-united. This was certainly well affected by the end of the fourth or beginning of the fifth week.

From the extension of the foot in the deformed state, there were two effects which required time, friction, and mechanical support to relieve after complete re-union of the tendons had taken place. 1st. The antagonistic power of the flexors of the foot was not entirely regained until the thirty-fifth or thirty-sixth day. The consequence of this was more or less trembling when it was in a flexed position. 2d. The integuments on the dorsum of the foot having like the flexor muscles been for years on the stretch, were, when the parts were restored to their natural position, wrinkled and relaxed. The effect of this upon the blood-vessels of the part, was to render them more tortuous in their course. Now, this condition of the blood vessels and the relaxed state of the integuments not affording the ordinary degree of mechanical support to the veins, subjected the foot to venous congestion whenever it was placed in a depending position. This effect ceased about the time when the flexor muscles regained their natural tension.

"In the beginning of the sixth week he began to walk, which at first was done very awkwardly, the limb operated on being very stiff at the ankle joint on account of the intermedium having adhered to the surrounding parts. From this time the spring shoe was put on with which he has been walking ever since. August the 21st, he informed me when his shoe was off, he could walk without limping in the slightest degree, and from this time (a little less than two months) I considered the cure perfect.

"In this case certainly all of the objects of the operation have been effected in a very short time.

"He never lost an hour's sleep during the process of the cure."

